# Supplementary figures and images for: Engrailed-2 promotes a malignant phenotype of esophageal squamous cell carcinoma through upregulating the expression of pro-oncogenic genes
Source: PeerJ. 2020 Feb 20;8:e8662. doi: 10.7717/peerj.8662 (PMC7036277; doi:10.7717/peerj.8662)

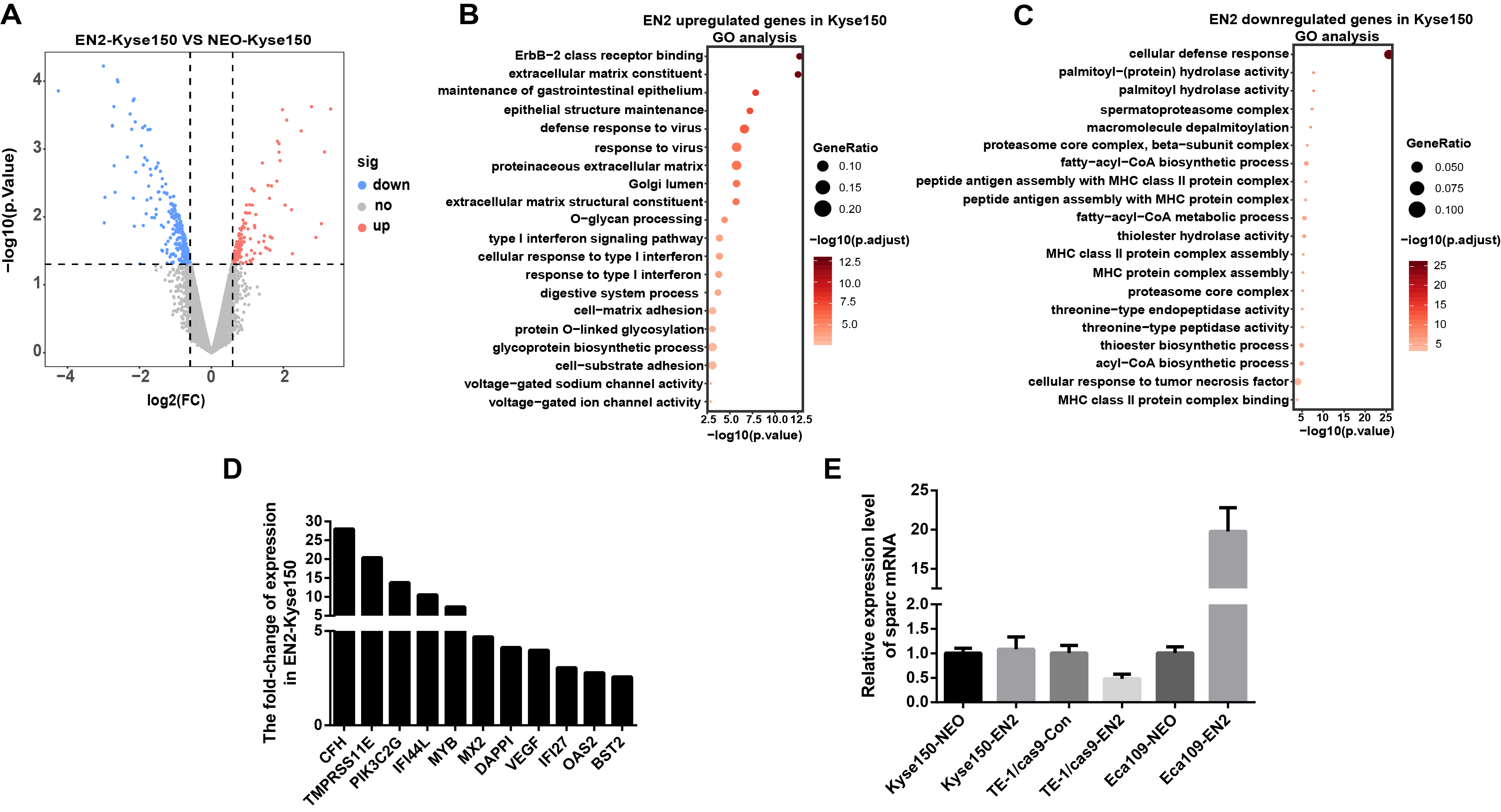

Supplement: Supplemental Information 1 — (A) Volcano maps show the differentially expressed genes in EN2-Kyse150 as compared with the NEO-Kyse150 cells. (B) Gene ontology (GO) analysis identified biological processes impacted by EN2 upregulated genes in Kyse150 cells. (C) GO analysis identified biological processes impacted by EN2 downregulated genes in Kyse150 cells. (D) Expression of the upregulated genes with high fold-change in EN2-Kyse150 and NEO-Kyse150 cells by means of qRT-PCR. (E) The mRNA expression level of SPARC in overexpression or silencing of EN2 of ESCC cell lines. [file peerj-08-8662-s001.png]

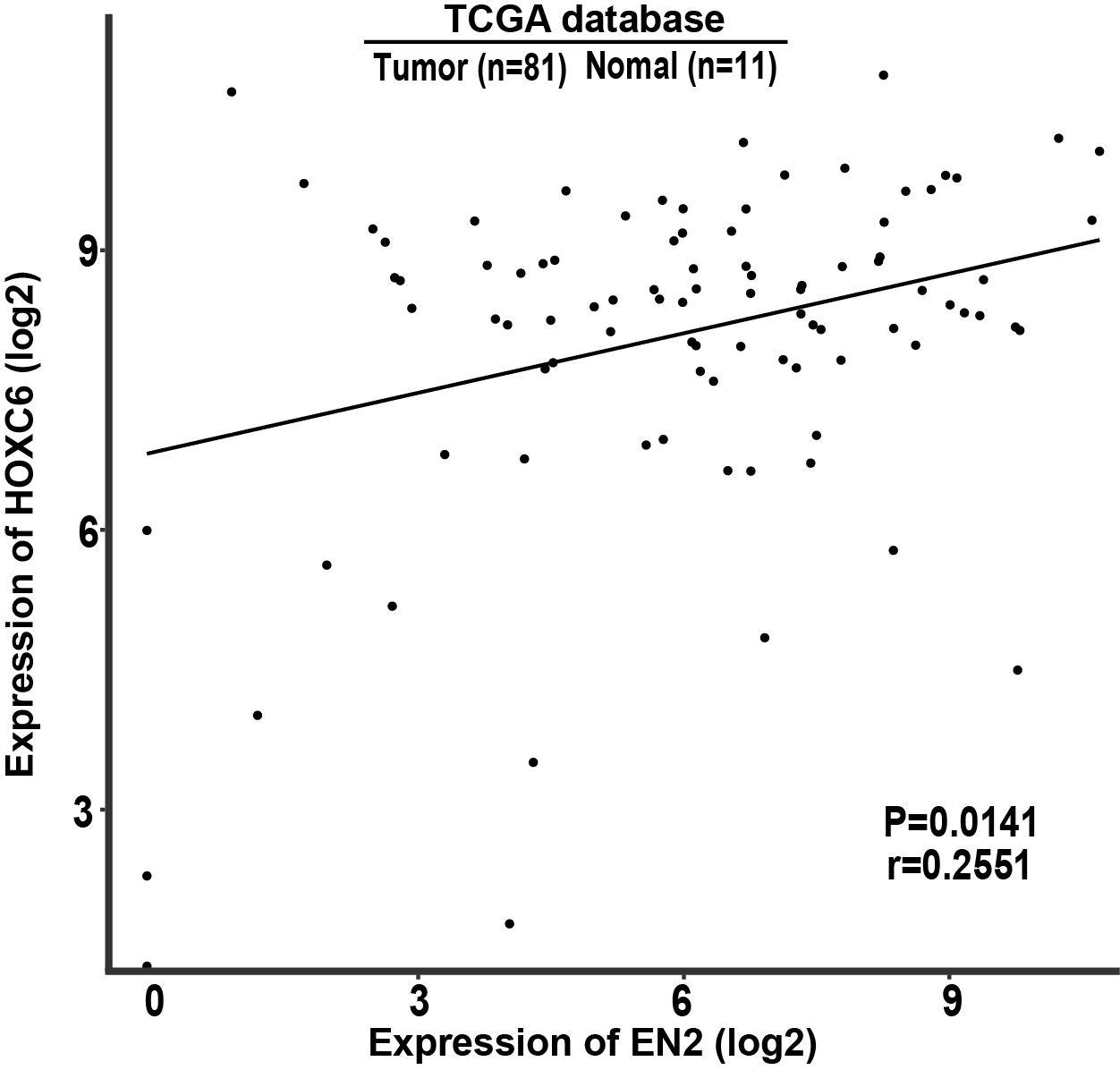

Supplement: Supplemental Information 2 [file peerj-08-8662-s002.png]
