# Supplementary material for: Engrailed-2 promotes a malignant phenotype of esophageal squamous cell carcinoma through upregulating the expression of pro-oncogenic genes
Source: PeerJ. 2020 Feb 20;8:e8662. doi: 10.7717/peerj.8662 (PMC7036277; doi:10.7717/peerj.8662)
Supplement: Supplemental Information 3 [file peerj-08-8662-s003.docx]

Supplementary material

Table S1. Sequences of the primers used in qRT-PCR

| **Genes** | **Sense (5' -3')** | **Antisense (5' -3')** | |  |
| --- | --- | --- | --- | --- |
| SPARC | CCCATTGGCGAGTTTGAGAAG | | CAAGGCCCGATGTAGTCCA | |
| AKR1C1 | TCCAGTGTCTGTAAAGCCAGG | | CCAGCAGTTTTCTCTGGTTGAA | |
| FRZB | GAGCCCATACTCATCAAGTACCG | | CCTCGGGAGAGATGCACAC | |
| AKR1C3 | GTCATCCGTATTTCAACCGGAG | | CCACCCATCGTTTGTCTCGTT | |
| KLRC2 | GCCAGCATTTTACCTTCCTCA | | ACTGCACAGTTAAGTTCAGCAT | |
| PLCL2 | TTCAGAACTCAAAAAGGTTCGCT | | GCTGCGGAATATGTCTGTGTT | |
| CRABP2 | CGCACCACAGAGATTAACTTC | | TCTGGTCCACGAGGTCTT | |
| ANXA10 | GCTGGCCTCATGTACCCAC | | CAAGCAGTAGGCTTCTCGC | |
| PTGS2 | ATGCTGACTATGGCTACAAAAGC | | TCGGGCAATCATCAGGCAC | |
| MCTP1 | AGTTTACGCCTATCAGACCTACA | | GATCGCTCAACCCGTTGGAAT | |
| DIO2 | TCCTCCTCGATGCCTACAAAC | | GTGAGTAGACCAGTAGTCTGCT | |
| EN2 | CCGGCGTGGGTCTACTGTA | | CCTCTTTGTTCGGGTTCTTCTT | |
| GAPDH | ATGCTGGCGCTGAGTACGTC | | GGTCATGAGTCCTTCCACGATA | |
